# Disuse‐induced muscle fibrosis, cellular senescence, and senescence‐associated secretory phenotype in older adults are alleviated during re‐ambulation with metformin pre‐treatment

**DOI:** 10.1111/acel.13936

**Published:** 2023-07-24

**Authors:** Jonathan J. Petrocelli, Alec I. McKenzie, Naomi M. M. P. de Hart, Paul T. Reidy, Ziad S. Mahmassani, Alexander R. Keeble, Katie L. Kaput, Matthew P. Wahl, Matthew T. Rondina, Robin L. Marcus, Corrine K. Welt, William L. Holland, Katsuhiko Funai, Christopher S. Fry, Micah J. Drummond

**Affiliations:** ^1^ Department of Physical Therapy and Athletic Training University of Utah Salt Lake City Utah USA; ^2^ Department of Nutrition and Integrative Physiology University of Utah Salt Lake City Utah USA; ^3^ Department of Kinesiology, Nutrition, and Health Miami University Oxford Ohio USA; ^4^ Molecular Medicine Program University of Utah Salt Lake City Utah USA; ^5^ Center for Muscle Biology University of Kentucky Lexington Kentucky USA; ^6^ Department of Internal Medicine University of Utah Salt Lake City Utah USA

**Keywords:** aging, atrophy, collagen, fibrosis, inflammation, metformin, SASP, senescence

## Abstract

Muscle inflammation and fibrosis underlie disuse‐related complications and may contribute to impaired muscle recovery in aging. Cellular senescence is an emerging link between inflammation, extracellular matrix (ECM) remodeling and poor muscle recovery after disuse. In rodents, metformin has been shown to prevent cellular senescence/senescent associated secretory phenotype (SASP), inflammation, and fibrosis making it a potentially practical therapeutic solution. Thus, the purpose of this study was to determine in older adults if metformin monotherapy during bed rest could reduce muscle fibrosis and cellular senescence/SASP during the re‐ambulation period. A two‐arm controlled trial was utilized in healthy male and female older adults (*n* = 20; BMI: <30, age: 60 years+) randomized into either placebo or metformin treatment during a two‐week run‐in and 5 days of bedrest followed by metformin withdrawal during 7 days of recovery. We found that metformin‐treated individuals had less type‐I myofiber atrophy during disuse, reduced pro‐inflammatory transcriptional profiles, and lower muscle collagen deposition during recovery. Collagen content and myofiber size corresponded to reduced whole muscle cellular senescence and SASP markers. Moreover, metformin treatment reduced primary muscle resident fibro‐adipogenic progenitors (FAPs) senescent markers and promoted a shift in fibroblast fate to be less myofibroblast‐like. Together, these results suggest that metformin pre‐treatment improved ECM remodeling after disuse in older adults by possibly altering cellular senescence and SASP in skeletal muscle and in FAPs.

## Graphical abstract

Metformin was provided to older adults 2 weeks prior and during 5‐days of bed rest and was withdrawn during re‐ambulation. Re‐ambulation after acute disuse in older adults treated with metformin resulted in decreased markers of cellular senescence and senescence associated secretory phenotype (SASP), a decrease in muscle fibro‐adipogenic progenitor (FAP) myofibroblast populations, and lower muscle fibrosis.

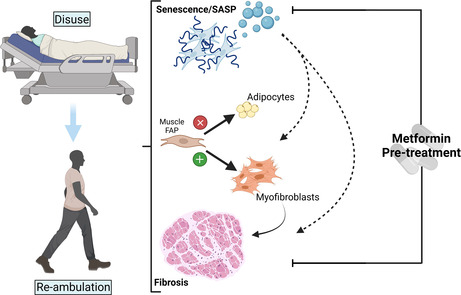

## 1 INTRODUCTION

Skeletal muscle disuse in older adults increases the risk of falls, hospitalization, and chronic disease development and accelerates age‐induced muscle loss (sarcopenia) (Kehler et al., 2019). Recovery of skeletal muscle function following disuse is subpar in older compared to younger individuals when given a similar recovery time frame, thus requiring more prolonged rehabilitation efforts to regain baseline function (Aagaard et al., 2010; Hvid et al., 2014; Pisot et al., 2016; Suetta et al., 2009; Suetta et al., 2013). Therefore, there is a need for translational therapeutic solutions targeting disuse atrophy and muscle recovery with aging.

Metformin is a commonly prescribed and cost‐effective drug used to lower blood glucose in individuals with type 2 diabetes mellitus. Recently, metformin has gained traction to be considered as an alternate use therapeutic in treating a broad range of age‐related diseases (Check Hayden, 2015; Kulkarni et al., 2020; Ng et al., 2014; Whittington et al., 2013), particularly fueled by the targeting aging with metformin (TAME) trial (Kulkarni et al., 2020). Metformin has also been shown to improve muscle regeneration following burn injury and reduce muscle atrophy in immobilized mice, which may be partly attributed to its regulation of muscle fibrosis (Wang et al., 2023; Yousuf et al., 2020). Moreover, we previously showed that metformin improved collagen remodeling and partially restored myofiber cross‐sectional area during re‐ambulation following hindlimb unloading in aged mice (Petrocelli et al., 2021). We surmise that during muscle regrowth following disuse atrophy, metformin may regulate collagen deposition through activation of macrophages and fibro‐adipogenic progenitors (FAPs) as metformin can directly modulate macrophage inflammatory activity (Cameron et al., 2016) and macrophages play an essential role in muscle extracellular matrix (ECM) remodeling (Dort et al., 2019; Stepien et al., 2020) through matrix metalloproteinase secretion and by regulating the accumulation and clearance of FAPs (Abramowitz et al., 2018; Sutherland et al., 2023). Indeed, the importance of macrophages in muscle remodeling is well understood as the absence of macrophages corresponds with muscle fibrosis and impaired muscle recovery (Arnold et al., 2007; Martinez et al., 2010; Ochoa et al., 2007) while in humans, muscle macrophage content positively correlates with muscle size after exercise training (Walton et al., 2019). Interventional therapy such as metformin is particularly relevant in this context since macrophage and FAP function are disrupted during recovery in aging (Fix et al., 2021; Lukjanenko et al., 2019; Reidy et al., 2019; Schuler et al., 2021).

Metformin is also well characterized to prevent cellular senescence and the senescence‐associated secretory phenotype (SASP) (Chen et al., 2023; Fang et al., 2018; Jadhav et al., 2013; Moiseeva et al., 2013). Senescent cells accumulating with advanced age have secretory profiles mainly containing cytokines, chemokines, ECM remodeling proteins, and growth factors (Englund et al., 2021). Indeed, FAPs are prone to senescence and were identified in aged skeletal muscle to display a high senescence phenotype (Zhang et al., 2022). The role of FAPs in ECM remodeling is critically important in muscle regeneration and regrowth, and recently the contribution of senescent cells to muscle atrophy has garnered attention. For instance, the whole body overexpression of senescent‐driving p21 protein resulted in skeletal muscle atrophy, fibrosis, and impaired physical function (Englund et al., 2022). We too have recently shown that myotube atrophy coincided with enhanced senescent cell abundance (Petrocelli et al., 2023). In aged individuals, skeletal muscle p21 expression is increased, while in aged mice, senescent muscle FAPs exhibit heightened inflammatory transcriptional pathways (Zhang et al., 2022). Therefore, it is reasonable that fibroblast senescence contributes to aberrant ECM remodeling thus dysregulating muscle recovery in aging. However, it is unknown if metformin can offset cellular senescence and modulate ECM remodeling during muscle recovery when given during muscle disuse in older adults.

Therefore, the purpose of this study was to determine the influence of metformin pre‐treatment on muscle ECM remodeling, macrophage content, and cellular senescence/SASP in older adults during bed rest, and a short‐term re‐ambulation period. We hypothesized that metformin would attenuate myofiber atrophy and cellular senescence/SASP and improve collagen remodeling during disuse and recovery in older adults.

## 2 METHODS

### 2.1 Subject characteristics and exclusion criteria

Healthy male and female older adults were recruited from the Salt Lake City area using local advertisements; subject characteristics can be found in Table 1. Exclusion criteria included cardiac, pulmonary, hepatic, vascular, hematologic, oncologic, and neurologic disease, as well as weight loss or dieting, diabetes (HbA1c >6.5), or use of other glucose‐lowering therapies. Patients with chronic kidney disease (serum creatinine >1.5 mg/dL) were excluded, and serum creatinine levels were monitored throughout bed rest. Eligibility blood screening (including a 2‐h oral glucose tolerance test (OGTT)) and inpatient procedures were performed at the University of Utah Center for Clinical and Translational Sciences (CCTS). In accordance with the Declaration of Helsinki, this study was approved by the University of Utah Institutional Review Board (IRB #93579) prior to any participant recruitment or data collection. Participants were informed that their participation was voluntary and that they may withdraw at any time for any reason. This trial received approval from the FDA (Investigational New Drug: IND 132366) and is registered on clinicaltrials.gov (NCT03107884).

**TABLE 1:** Subject characteristics.

	Placebo	Metformin
Female/male	6/4	5/5
Age (years)	66 ± 4.5	71.7 ± 5.1
Height (cm)	168.8 ± 13.5	169.4 ± 10.1
Weight (kg)	77.5 ± 18.1	78.8 ± 17.6
BMI (kg/m^2^)	27.0 ± 3.9	27.4 ± 5.0
Glucose (mg)	94.8 ± 8.2	93.4 ± 9.6
HbA1c (%)	5.5 ± 0.3	5.6 ± 0.3

*Note*: Data are mean ± SD.

### 2.2 Treatment allocation

Using a double‐blind, placebo‐controlled study design, older adults were randomized to either placebo or metformin groups and were sex‐matched. Randomization was performed by an individual who was not involved in the experimental implementation or data analyses. Treatment allocation was performed using sequentially labeled envelopes, opened after verifying participants' eligibility. Metformin and placebo control pills were encapsulated to prevent the visual identification of the treatment assignment.

### 2.3 Trial design

**FIGURE 1:**
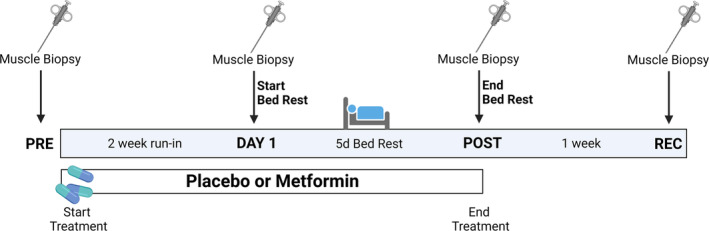
Experimental schematic. A muscle biopsy was taken prior to the beginning of any treatment (PRE) or intervention. Participants were then given placebo or metformin for 2 weeks and stopped taking their treatments 2 days before the start of bed rest. At the start of the first day of bed rest a muscle biopsy was taken (DAY 1), and participants began treatments again for the next 4 days of bed rest. On the 5th day of bed rest (POST), a muscle biopsy was taken, and participants stopped their treatments the night before and did not continue on their treatments for the remainder of the study. After a 1‐week recovery period (REC) participants returned and underwent another muscle biopsy.

Figure 1 displays the trial design and specifies when each study procedure occurred. Briefly, after the screening, participants underwent a muscle biopsy, and magnetic resonance imaging (MRI) before intervention (PRE). Then, they began either placebo or metformin during a 2‐week run‐in period. After 2 weeks, participants underwent a muscle biopsy on the first day of bed rest (DAY1), followed by 5 days of bed rest, continuing placebo/metformin during bed rest. After 5 days of bed rest (POST), participants underwent a muscle biopsy and MRI. Lastly, patients ceased placebo/metformin treatments and underwent a 7‐day re‐ambulation period (REC) followed by a final muscle biopsy. Metformin dosage began at 1 g/day working up to a dose of 2 g/day during the Run‐in period and remained at 2 g/day during the bed rest period (last dose on evening of bed rest Day 4). Further details of the metformin dosing regimen, bed rest safety measures, muscle biopsy procedures, and MRI analysis can be found in the Supplemental Methods.

### 2.4 Muscle biopsies

After a standardized dinner and overnight fast (at least 10 h), vastus lateralis percutaneous muscle biopsies were performed on the leg at PRE, DAY1, POST, and REC using the Bergstrom needle approach with manual suction (Evans et al., 1982). The biopsy site was marked with a skin marker 10–15 cm proximal to the patella, and the biopsy area cleaned with betadine and collected following administration of local anesthetic (1% lidocaine). Repeated biopsies were conducted 3 cm from prior incision sites. Muscle collected from the biopsy was dissected of non‐muscle tissue, blotted on sterile gauze to remove blood, and then allocated into analyses for immunohistochemistry (IHC), gene expression, and primary cell isolation. Tissue allocated for gene expression was washed with sterile, room temperature saline, immediately snap‐frozen in liquid nitrogen, and then stored at −80°C. IHC samples were selected based upon clear identification of muscle fiber directionality present in the sample and was blotted but not washed with saline (to prevent osmotic fiber size changes). IHC samples were mounted on an aluminum covered piece of cork with optimal cutting temperature (OCT) compound, placed in a metal container containing liquid nitrogen‐cooled isopentane for 20–30 s, and then kept on dry ice until storage at −80°C.

### 2.5 Bed rest

Total caloric intake during bed rest was predetermined by the research dietitian using the Harris‐Benedict equation adjusted for no physical activity. Daily caloric intake was evenly distributed over three meals, at hours 08:00, 13:00, and 18:00. Participants could consume water and noncaloric flavored beverages ad libitum throughout bed rest. Bed rest procedures were performed at the CCTS and conducted as previously reported (Reidy et al., 2017; Tanner et al., 2015). The participants were allowed to adjust the bed angle to read, eat, browse the internet, or watch television. Otherwise, they spent most of the inactivity period lying flat to sleep. Bathing and hygiene activities were performed at the sink in the hospital room while the participant was seated in a wheelchair. When needed, the toilet was accessed via the wheelchair and with assistance from the CCTS nursing staff. Adherence to bed rest was monitored 24 h per day by the CCTS nursing staff.

### 2.6 Immunofluorescence and histochemistry

Samples embedded in OCT compound on a cork wrapped in aluminum foil from each participant at PRE, DAY1, POST, and REC were removed from the cork at −25°C in a Leica CM1860 cryostat (Leica Biosystems) and sectioned at 10 μm. Sections were air‐dried at room temperature overnight and then stored in a slide box at −20°C until stained. Full details of all immunofluorescent and histochemical methods and reagents can be found in the Supplemental Methods.

### 2.7 Bulk RNA‐Sequencing and qPCR

Total RNA was isolated by homogenizing 10–20 mg of tissue in Qiazol Lysis Reagent (Qiagen, cat# 79306). The RNA was separated and precipitated using chloroform and isopropanol. Extracted RNA was washed with ethanol and then suspended in nuclease‐free water. RNA concentration was determined using an EPOCH (Take3, BioTek) spectrophotometer. Libraries were prepared with Illumina Stranded Total RNA Library Prep NEB Ultra II directional RNA library prep with rRNA depletion and RNA was sequenced using Illumina NovaSeq S4 Reagent Kit v1.5 150 × 150 bp Sequencing (100 M read pairs). Differentially expressed genes were identified using a 5% false discovery rate with DESeq2 version 1.30.00 and the hciR package. Data can be found on the Gene Expression Omnibus (GSE224900). KEGG, and REACTOME pathways were identified using the fast gene set enrichment analysis in MSigDB using a 10% FDR. Real‐time quantitative PCR was carried out with a CFX Connect real‐time PCR cycler (Bio‐Rad) following the manufacturers protocol for SYBR Green custom‐designed primers. All customer primers utilized were ordered from Bio‐Rad. Cycle threshold values of target genes were normalized to GAPDH gene expression, and then fold change values were calculated (ΔΔCT) with the respective PRE bed rest values as reference.

### 2.8 Primary myoblast and fibro‐adipogenic progenitor cell isolation and culture

Primary myoblasts and fibro‐adipogenic progenitor (FAP) cells were isolated from approximately 30–50 mg of fresh muscle tissue. Muscle tissue was minced in low glucose Dulbecco's Modified Eagle's Medium (DMEM) with 1% penicillin–streptomycin, followed by two washes in Hank's Balanced Salt Solution without Ca^2+^ and Mg^2+^. Tissue was then digested with collagenase II and trypsin in 1x phosphate‐buffered saline (PBS) for 30 min in a 37°C water bath. Digested muscle was then plated for 2 h in a 37°C, 5% CO_2_ incubator on a 60 mm tissue culture‐treated dish to adhere progenitor fibroblast populations. After 2 h, digested muscle was then plated on a collagen coated dish to adhere primary myoblast populations. On Passage 3, cells were plated and allowed to proliferate for 2 days in low glucose DMEM with 1% penicillin–streptomycin and 10% fetal bovine serum before collection. FAPs were stained with transcription factor 4/transcription factor 7 like 2 (TCF4/TCF7L2) to confirm fibroblast lineage.

### 2.9 Statistical analysis

Subject characteristics are reported as means ± standard deviation (SD); all other figures are means ± standard error (SE) with individual data points displayed. Data are reported as individual baseline (PRE) values linked to after bed rest (POST) or recovery (REC) values for a given participant or as a difference (Δ) from baseline values. To determine the interaction and main effects of treatment (placebo or metformin) by intervention days (time), a two‐way analysis of variance (ANOVA) was utilized with Sídák's post‐hoc analysis to determine significant differences within timepoints. Main effects of time, treatment, or interaction are provided above figure panels. Pearson correlations were used to identify associations between dependent measurements. For all statistical comparisons, significance was set at the level of *p* < 0.05. All analysis and figures were done with GraphPad Prism 9 software (La Jolla, CA, USA).

## 3 RESULTS

### 3.1 Metformin prevented bed rest‐induced myofiber atrophy in a fiber‐type‐dependent manner and reduced hybrid fibers

**FIGURE 2:**
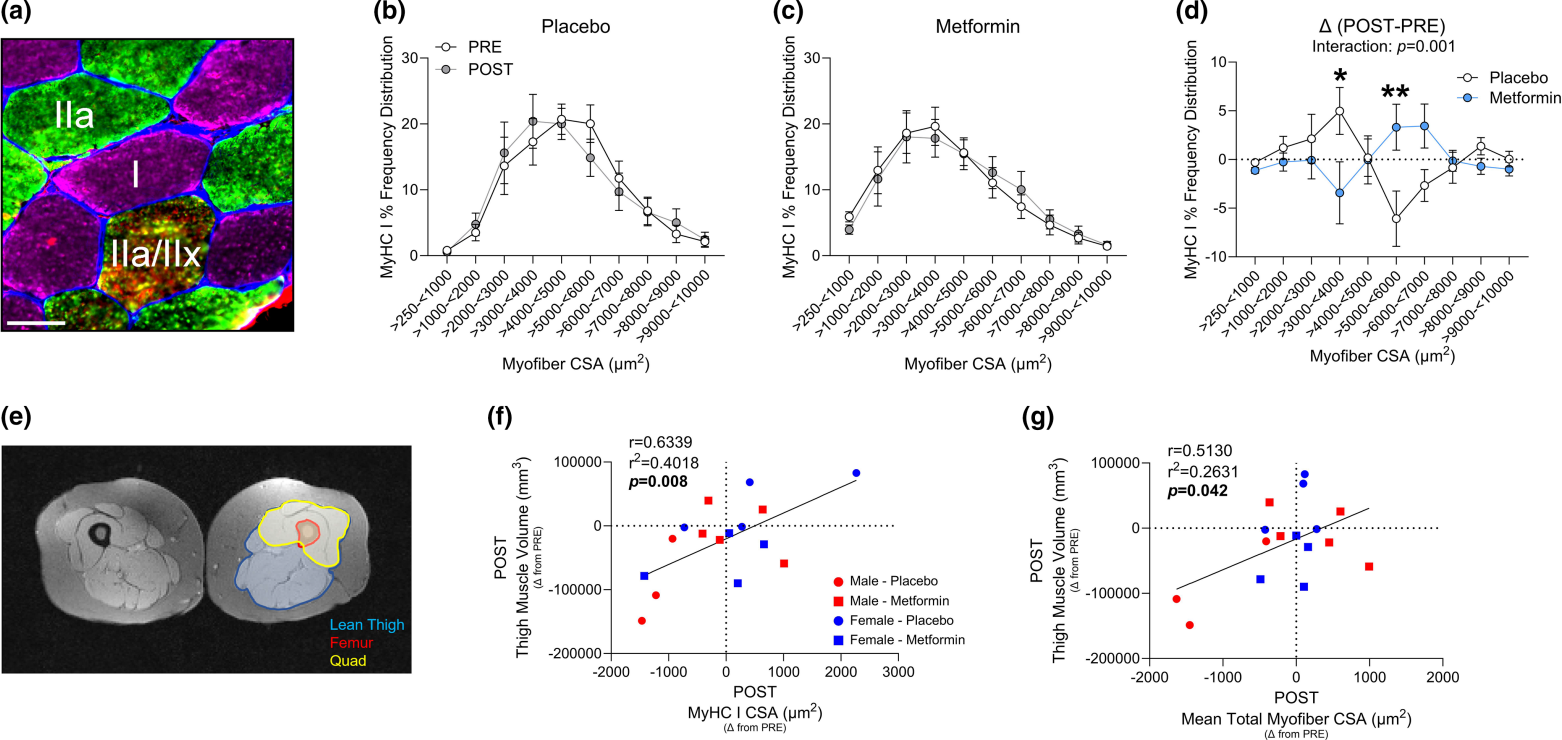
Metformin prevented myofiber cross‐sectional area reduction after bed rest. (a) Representative image used to determine myofiber type specific CSA. (b, c) Frequency distribution of myosin heavy chain I (MyHC I) PRE and POST. (d) POST delta (Δ) from PRE‐intervention percent fiber frequency of MyHC I myofiber cross‐sectional area (CSA) from 250 to 10,000 μm^2^. (e) Representative MRI image used to determine thigh muscle volume. (f) Correlation of the change in thigh muscle volume (mm^3^) after bed rest to the change in MyHC I myofiber CSA (μm^2^) after bed rest. (g) Correlation of the change in thigh muscle volume (mm^3^) after bed rest to the change in mean myofiber CSA (μm^2^) after bed rest. *p < 0.05. *N* = 7–10/group. Scale bar is 50 μm. Myofiber counts as mean ± SE: Placebo PRE: 555 ± 96, POST: 550 ± 53. Metformin PRE: 492 ± 68, POST: 435 ± 55.

A representative image used for myosin heavy chain (MyHC) cross‐sectional area (CSA) and fiber typing analyses can be found in Figure 2a. Mean total myofiber CSA after the 2‐week run‐in period (DAY 1), 5 days of bed rest (POST), or following 7 days of recovery (REC) was not different between groups (Figure S1a–i). However, the metformin‐treated group demonstrated fewer smaller (>3000–<4000 μm^2^) and greater larger (>5000–<6000 μm^2^; Figure 2b–d) MyHC I fibers after 5 days of bed rest and after the 2‐week run‐in (Figure S1j–l) compared to Placebo, with no differences at REC (Figure S1m–o). MyHC IIa CSA was not different between the two groups at any of the time points (Figure S1p–x). To validate that myofiber CSA changes were associated to whole muscle changes, we correlated the post bed rest difference in MyHC I and mean total myofiber CSA to the difference in thigh muscle volume measured via MRI and observed a positive relationship (Figure 2e–g).

MyHC fiber distribution displayed MyHC I as the most abundant (Figure S2a) and largest fiber population in both groups (Figure S2b,c). Percent MyHC I (Figure S2d) and MyHC IIa (Figure S2e) content were unaltered by bed rest or metformin treatment. However, MyHC I & IIa hybrids were lower in the metformin group throughout the intervention (Figure S2f). Both groups decreased MyHC I & IIa hybrids after bed rest (Figure S2g), and tended (*p* = 0.08) to be lower after recovery (Figure S2h). MyHC IIa & IIx hybrids displayed a trend to decrease with metformin treatment (Figure S2i), and when combining all hybrid fiber types, an effect for metformin to reduce total hybrid fibers was evident (Figure S2j) only after recovery (Figure S2k,l).

### 3.2 Muscle macrophages, but not satellite cells or capillaries, dynamically change during bed rest and recovery

**FIGURE 3:**
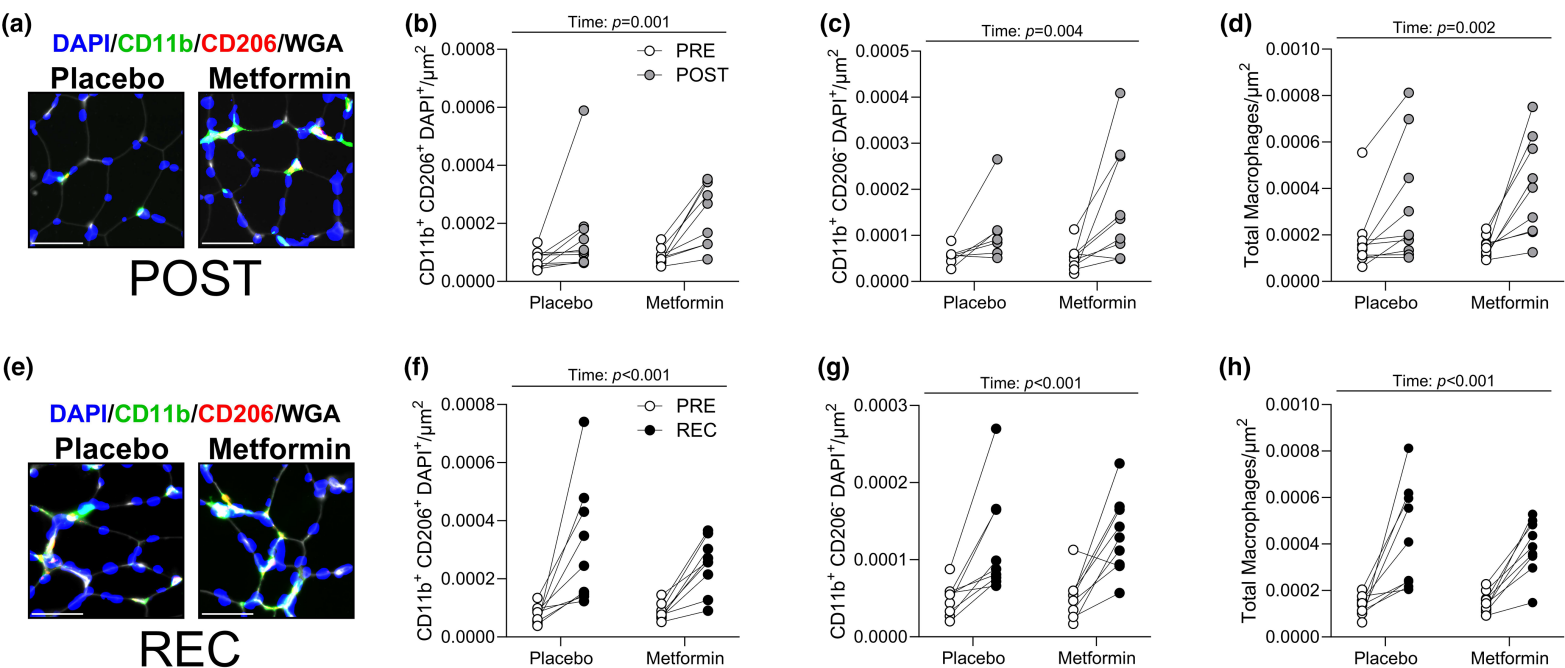
Macrophage content increased after bed rest and during recovery. (a) After bed rest representative image used to identify CD11b^+^ and CD206^+^ cells. (b) CD11b^+^ and CD206^+^ (anti‐inflammatory‐like) cells after bed rest. (c) CD11b^+^ and CD206^−^ (pro‐inflammatory‐like) cells after bed rest. (d) All labeled cells (total macrophages) after bed rest. (e) After recovery representative image used to identify CD11b^+^ and CD206^+^. (f) CD11b^+^ and CD206^+^ cells after recovery. (g) CD11b^+^ and CD206^−^ cells after recovery. (h) All labeled cells after recovery. *N* = 7–10/group. Scale bar is 50 μm.

We next examined the abundance of satellite cells, capillaries, and macrophages as these cells/tissues are well‐known for participating in skeletal muscle remodeling and are disrupted in aging (Larsson et al., 2019). Representative images of satellite cells and capillaries after bed rest are shown in Figure S3a. Total satellite cell content was reduced in the metformin group (Figure S3b) in both MyHC I & II fibers (Figure S3c–e). Total capillary content was not different between groups (Figure S3f). To assess macrophage populations, integrin subunit alpha m (CD11b) and mannose receptor C‐type (CD206) positive cells were observed after bed rest (representative images are shown in Figure 3a). After bed rest, CD11b and CD206 positive cells (anti‐inflammatory‐like; Figure 3b), CD11b positive/CD206 negative cells (pro‐inflammatory‐like; Figure 3c), and total macrophages (Figure 3d) were increased in both groups. Macrophage populations remained elevated after recovery in both groups (Figure 3e–h).

### 3.3 Metformin altered collagen content and architecture during recovery following bed rest

**FIGURE 4:**
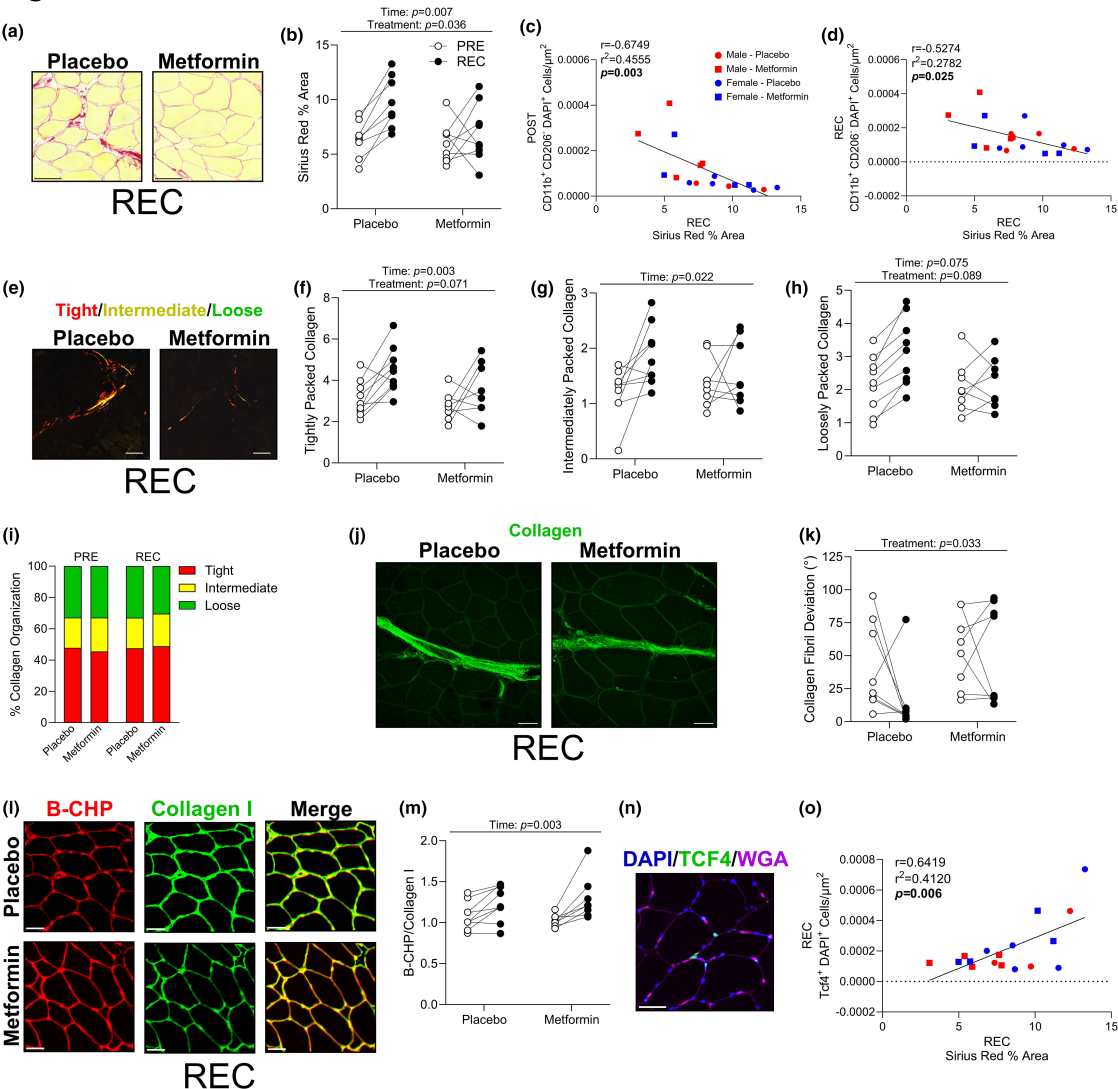
Metformin reduced collagen deposition and improved collagen remodeling. (a) Representative images of Sirius Red staining after recovery. (b) Sirius Red percent area after recovery. (c) Correlation of CD11b^+^ CD206^−^ cells after bed rest to Sirius Red percent area after recovery. (d) Correlation of CD11b^+^ CD206^−^ cells after recovery to Sirius Red percent area after recovery. (e) Representative images of Sirius Red after polarized light exposure after recovery. (f–h) Tightly, intermediately, and loosely packed collagen content after recovery. (i) Percent organization of tight, intermediate, and loose packed collagen. (j) Representative image of second harmonic generation imaging after recovery. (k) Collagen fibril deviation degree after recovery. (l) Representative images of biotinylated‐hybridizing peptide (B‐CHP), collagen I, and merged images after recovery. (m) Ratio of B‐CHP/Collagen I ratio after recovery, as a surrogate marker for collagen turnover. (n) Representative image used to identify Tcf4^+^ cells. (o) Correlation of Tcf4^+^ cells after recovery to Sirius Red % area after recovery. *N* = 8–10/group. Scale bars are 50 μm.

Given the role of macrophages in skeletal muscle fibroblast and ECM remodeling (Abramowitz et al., 2018; Dort et al., 2019), we quantified collagen deposition, collagen turnover, and fibroblast content. Representative images of Sirius Red staining following the recovery can be found in Figure 4a. After recovery, metformin prevented the increase in Sirius Red percent area observed in the placebo‐treated group (Figure 4b). Interestingly, the number of CD11b positive, CD206 negative cells identified after bed rest and recovery were negatively correlated with the change in Sirius Red after recovery (Figure 4c,d). This may support that the enhanced macrophage content after bed rest contributed to the total reduction in collagen deposition during recovery. We observed aspects of collagen architecture and noted a trend that tightly and loosely packed collagen were each increased in the placebo group after recovery (Figure 4e–h). This was likely due to the increase in collagen content, as when normalized to total collagen the composition of collagen packing was unaltered (Figure 4i). Using second harmonic generation imaging (Figure 4j), the placebo group had a decreased average collagen fibril deviation after recovery (Figure 4k), indicative of more parallel collagen alignment. As a surrogate marker of collagen turnover, we utilized a biotinylated collagen hybridizing peptide (B‐CHP) that interacts with unfolded collagen and is co‐stained with collagen I. We found that the B‐CHP/collagen I ratio (collagen turnover; representative images found in Figure 4l) was increased in both groups (Figure 4m). Total fibroblast content (Tcf4+ cells; representative image in Figure 4n) was not different between groups (data not shown). However, Tcf4+ cells positively correlated with the change in Sirius Red deposition during recovery (Figure 4o), possibly inferring a role for metformin to act on fibroblasts during recovery.

### 3.4 Muscle recovery was characterized by an enhanced collagen transcriptional profile and increased senescence‐associated secretory phenotype factors that were prevented with metformin

**FIGURE 5:**
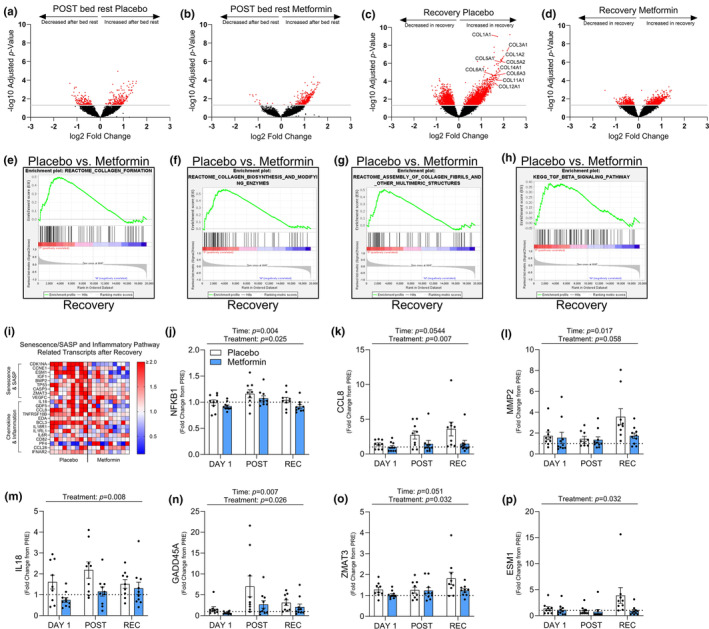
Collagen transcriptional profiles and cellular senescence driving transcripts are reduced with metformin. After bed rest volcano plot displaying significantly up and downregulated transcripts in red with placebo (a) or metformin (b). After recovery volcano plot displaying significantly up and downregulated transcripts in red with placebo (c) or metformin (d), with collagen gene family members highlighted. (e–h) GSEA enrichment plots for Collagen Formation, Collagen Biosynthesis, and Modifying Enzymes, Assembly of Collagen Fibrils and Other Multimeric Structures, and TGF‐β Signaling pathways after recovery in placebo versus metformin (i) Heat map of senescence/SASP and inflammatory related transcripts after recovery as a fold change from PRE. (j–p) Transcriptional changes of cellular senescence driving transcripts as a fold change from PRE, at DAY1, POST, and REC. Log10 Adjusted *p*‐value of 1.3 (*p* < 0.05) was used to determine significance for volcano plots. *N* = 9–10/group.

To further examine the underlying mechanisms of altered muscle remodeling programs in the metformin treated group during recovery from bed rest, we conducted RNA sequencing on muscle biopsy samples at PRE, POST, and REC. After bed rest, 441 gene transcripts changed in the placebo group (Figure 5a), whereas in the bed rest metformin group, 377 genes were altered (Figure 5b). After 7 days of recovery, the placebo group displayed 3550 changed gene transcripts with collagen isoforms observed as the highly upregulated transcripts (Figure 5c) whereas, this transcriptional phenotype was remarkably blunted in the metformin group (1386 changed transcripts; Figure 5d). Indeed, transcriptional pathways related to collagen biosynthesis, assembly, and fibrosis after recovery were enriched in the placebo versus the metformin group (Figure 5e–h). We also saw an increase in transcripts related to cellular senescence, the senescence‐associated secretory phenotype (SASP), and inflammatory signaling that was higher in the placebo, but not in the metformin group after recovery (Figure 5i). Several of these SASP transcripts that were different between the groups have been described as inducers and reinforcers of the senescence program in skeletal muscle (Englund et al., 2022) (Figure 5j–p).

### 3.5 Metformin reduced cellular senescence markers in muscle tissue and skeletal muscle FAPs

**FIGURE 6:**
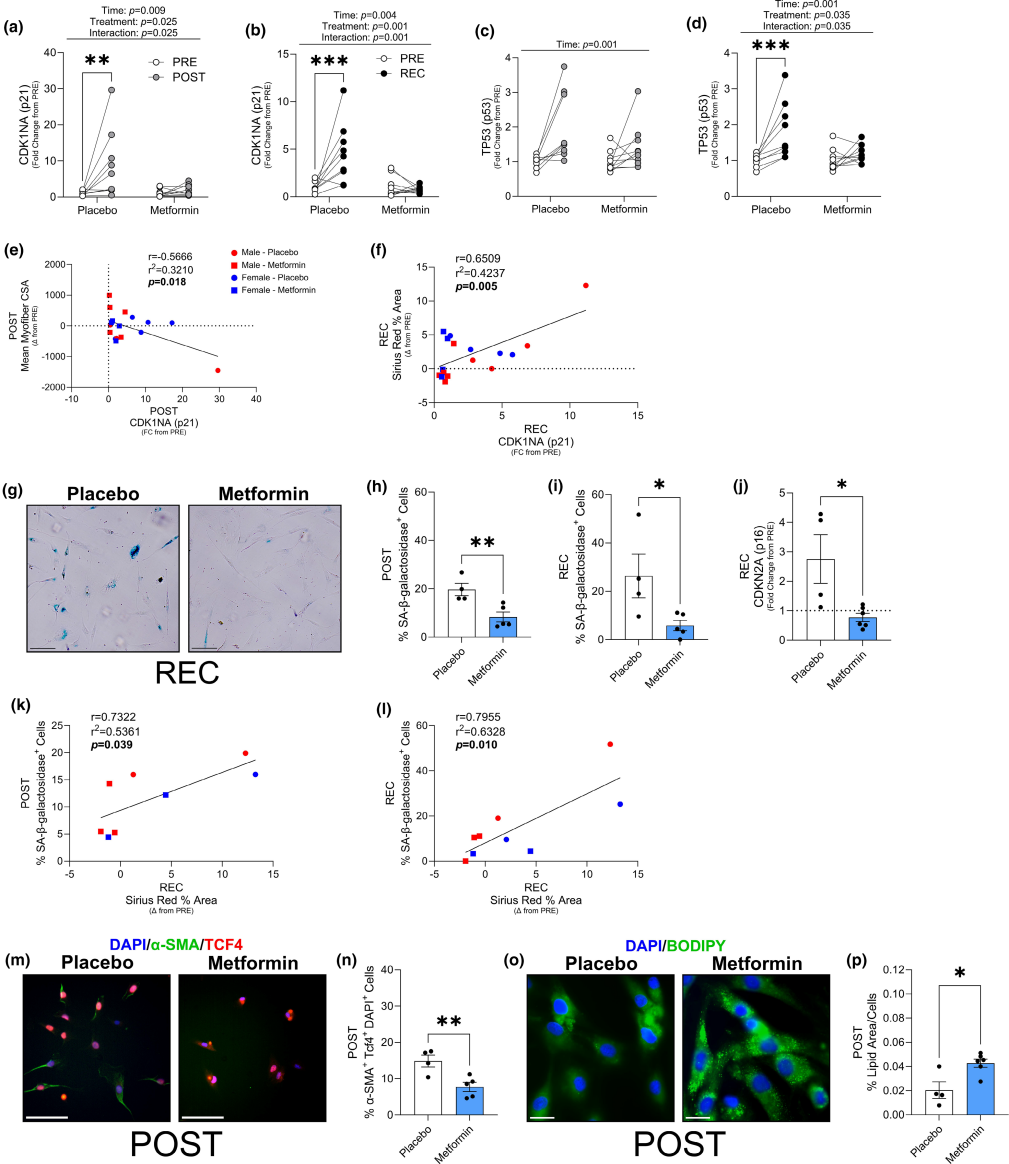
Metformin decreased markers of cellular senescence in whole muscle and muscle resident fibro‐adipogenic progenitors (FAPs). CDK1NA (p21) transcription as a fold change from PRE, after bed rest (a) and after recovery (b). TP53 (p53) transcription as a fold change from PRE, after bed rest (c) and after recovery (d). (e) Correlation of mean myofiber area after bed rest to the fold change in p21 transcription after bed rest. (f) Correlation of Sirius Red percent area after recovery to the fold change in p21 transcription after recovery. (g) Representative images of senescent associated (SA)‐β‐galactosidase^+^ muscle resident FAPs after recovery. Percent SA‐β‐galactosidase^+^ muscle resident FAPs after bed rest (h) and after recovery (i). (j) CDKN2A (p16) gene expression in muscle resident FAPs. (k) Correlation of SA‐β‐galactosidase^+^ to the change in Sirius Red percent area after bed rest and (l) recovery. (m) Representative image of after bed rest α‐smooth muscle Actin (α‐SMA) and TCF4^+^ cells. (n) Percent α‐SMA and TCF4^+^ cells after bed rest. (o) Representative image of after bed rest BODIPY (lipid droplets) staining. (p) Percent lipid droplets after bed rest. ***p < 0.001, **p < 0.01, *p < 0.05. *N* = 9–10/group for transcriptional data. *N* = 4–5/group for muscle resident FAP analysis. Scale bar is 50 μm for SA‐β‐galactosidase, 100 μm for α‐SMA, and 25 μm for BODIPY stained images.

The classical senescence markers, cyclin dependent kinase inhibitor 1a (CDK1NA; p21) and tumor protein p53 (TP53; p53) (Englund et al., 2021) transcripts in whole muscle were increased in the placebo, but not metformin group after bed rest and recovery (Figure 6a–d). Interestingly, the change in myofiber CSA was negatively correlated with the change in p21 whole muscle transcripts after bed rest (Figure 6e), while the change in Sirius Red percent area was positively correlated to the change in p21 transcripts after recovery (Figure 6f). We isolated primary muscle progenitor cells and fibro‐adipogenic progenitor cells from whole muscle biopsies to determine if the senescent cell signature may be partly arising from these cell types. Senescent markers, p21 and p16 in primary muscle progenitor cells isolated from muscle biopsy samples were not different between placebo and metformin groups at recovery (Figure S4a,b). This data suggests that metformin‐mediated reduction in whole muscle cellular senescence/SASP and fibrosis are independent of changes in myoblast cellular senescence profile. Thus, we next decided to examine if skeletal muscle derived FAPs from these participants similarly exhibit a senescent phenotype. Indeed, senescence associated (SA)‐β‐galactosidase positive FAPs were decreased in the metformin group after bed rest and recovery (Figure 6g–i). After recovery, these cells also exhibited increased p16 gene expression in the Placebo group (Figure 6j) which is consistent with a previous report supporting enhanced senescent profiles in aged FAPs expressing p16 (Zhang et al., 2022). Interestingly, SA‐β‐galactosidase positive FAP cells after bed rest (Figure 6k) or recovery (Figure 6l), positively correlated with the change in whole muscle Sirius Red content from pre to recovery, indicative that FAP senescence may alter collagen deposition. To determine if the increased senescent markers coincided with a change in FAP fate, we stained the cells for the myofibroblast marker, alpha‐smooth muscle actin (α‐SMA) (Figure 6m,n) and lipid droplet content (BODIPY; Figure 6o,p). After bed rest, the metformin‐treated group had less α‐SMA positive cells (Figure 6n) and increased lipid droplet area (Figure 6p), potentially indicating that metformin may have balanced the fate of FAPs with lower myofibroblast‐like and higher adipocyte‐like populations in vivo.

## 4 DISCUSSION

In this randomized placebo‐controlled trial, we provided glucose‐tolerant older adults with a clinical dose of metformin during bed rest to capitalize on the senomorphic properties of metformin in order to improve collagen remodeling and cellular senescence during disuse and recovery. The major findings from this study were that metformin‐treated individuals were characterized by higher type I myofiber CSA size after bed rest and less muscle fibrosis during recovery corresponding to reduced markers of senescence/SASP in whole muscle and isolated human primary muscle FAPs. We also observed a positive correlation of whole muscle p21 transcription as well as SA‐β‐galactosidase+ FAP cells with muscle collagen deposition after recovery, suggesting that cellular senescence in whole muscle and FAPs may be related to aberrant collagen remodeling events. Together, pre‐loading with metformin may alleviate excess muscle collagen deposition during recovery from disuse in aging by reducing skeletal muscle and FAP senescence/SASP.

A primary finding from this study was that metformin prevented bed rest induced myofiber‐type specific cross‐sectional area loss. Metformin has previously shown preferential effects toward oxidative fibers to prevent slow‐to‐fast switching during disuse atrophy in rats (Sharlo et al., 2022). While we did not observe major shifts in myofiber composition after bed rest or recovery, we did indicate MyHC I‐specific atrophy protection and a reduction in hybrid fibers with metformin treatment. The preference of metformin to possibly impact slow, oxidative fibers likely stems from the well characterized activation of AMPKα (5′AMP‐activated protein kinase alpha) by metformin. AMPKα protein expression is highest in slow‐twitch muscle and AMPKα deletion promotes fast‐twitch myofiber composition (Schiaffino & Reggiani, 2011). Additionally, we observed MyHC I to be the most abundant isoform in the vastus lateralis of these older individuals also likely contributing to the more robust effect in this myofiber type.

Metformin has been well described to have anti‐inflammatory properties, specifically through inhibition of nuclear factor kappa B (NF‐κB) signaling (Kulkarni et al., 2020). Though we are unsure the specific cell types affected by the anti‐inflammatory properties of metformin, we observed that macrophage content measured after bed rest or during re‐ambulation was negatively correlated to collagen content at recovery, suggesting that an increase in muscle macrophage content could be related to lower collagen deposition during muscle regrowth following disuse atrophy. Metformin has been shown to enhance immune function (Foretz et al., 2023; Justice et al., 2021; Nojima & Wada, 2023) possibly by directly modulating macrophage function and inflammatory profile (Cameron et al., 2016; Qing et al., 2019) which together may exert downstream events that affect ECM remodeling. Indeed, the subjects treated with metformin had lower collagen content, higher collagen remodeling, and less aligned collagen fibrils suggestive of a more permissive matrix to support muscle fiber regrowth (Brashear et al., 2022). We acknowledge that only macrophage content was assessed in this study thus perhaps macrophage function (e.g., phagocytosis) may have differed between placebo and metformin groups. It is also possible that we may have missed a group difference in muscle macrophage content at an earlier time point in recovery. Indeed, muscle size was fully restored in both groups by 7‐days recovery further suggesting that acute muscle intracellular events likely had already transpired. Together, metformin reduced pro‐inflammatory transcriptional profiles which corresponded to improved collagen remodeling during recovery.

In coordination with macrophages, FAPs play an essential role in the proper remodeling of skeletal muscle following injury (Abramowitz et al., 2018) yet are functionally impaired with aging (Fix et al., 2021; Lukjanenko et al., 2019; Reidy et al., 2019; Schuler et al., 2021). While the necessity of FAPs for full resolution from muscle injury is well understood (Murphy et al., 2011), less is known about FAP senescence in muscle repair and remodeling after disuse, especially in aged adults. An important observation we noted in this study was that metformin‐treated older adults had an overall lower level of whole muscle transcriptional markers of senescence (e.g., p21, p53). Therefore, we examined muscle FAPs isolated and cultured from subjects at bed rest and recovery and confirmed that the decreased senescent program in response to metformin at least partly stemmed from muscle FAPs. Interestingly, senescent FAP content positively correlated with whole muscle collagen deposition after recovery, indicating that FAP senescence may alter ECM remodeling functions. Many SASP factors are also established chemokines and ECM remodeling proteins (matrix metalloproteinases, transforming growth factor β family), while global p21 overexpression corresponded with increased skeletal muscle collagen deposition (Englund et al., 2022). Likewise, aged mice are characterized with increased senescent FAPs (Zhang et al., 2022), possibly linking increased senescent cell burden with aging and muscle fibrosis. In addition to a possible modulatory role on whole muscle anti‐inflammatory profiles by metformin during bed rest and recovery, we suggest that metformin could impact FAPs directly/indirectly by reducing cellular senescence/SASP (Fang et al., 2018; Han et al., 2016; Moiseeva et al., 2013). Others have observed metformin to reduce cellular senescence in FAPs through upregulation of antioxidant pathways (Fang et al., 2018), anti‐inflammatory properties via downregulation of NF‐κB translocation (Moiseeva et al., 2013), and through activation of AMPKα signaling (Han et al., 2016). Thus, the seno‐therapeutic properties of metformin, particularly on muscle resident FAPs, may underlie improved collagen remodeling in older adults during recovery from bed rest.

In summary, metformin treatment 2 weeks before and during disuse improved older adult myofiber remodeling during early re‐ambulation through alterations in collagen deposition. The effects of metformin on collagen deposition may be related to increased anti‐inflammatory transcriptional programs and reduced whole muscle and muscle resident FAP senescence/SASP. We conclude that short term delivery of metformin may be beneficial to ameliorate muscle atrophy and improve muscle recovery during short‐term recovery following disuse in older adults by targeting muscle cellular senescence/SASP.

## AUTHOR CONTRIBUTIONS

Experiments and data analysis were conducted by Jonathan J. Petrocelli, Alec I. McKenzie, Naomi MMP. de Hart, Paul T. Reidy, Ziad S. Mahmassani, Alexander R. Keeble, Katie L. Kaput, and Christopher S. Fry. Clinical data and procedures were performed or overseen by Matthew P. Wahl, Matthew T. Rondina, Corrine K. Welt, and Micah J. Drummond. Experimental design was conducted by Jonathan J. Petrocelli, Alec I. McKenzie, Paul T. Reidy, Matthew T. Rondina, Robin L. Marcus, William L. Holland, Katsuhiko Funai, and Micah J. Drummond. The manuscript was prepared by Jonathan J. Petrocelli, Alec I. McKenzie, and Micah J. Drummond. All authors contributed to editing the manuscript.

## FUNDING INFORMATION

Funding for this project was provided by NIA Grant (F99AG073493) awarded to JJP, NIA Grant (R21AG064576) and University of Utah Center on Aging pilot award to MJD. The content is solely the responsibility of the authors and does not necessarily represent the official views of the National Institutes of Health.

## CONFLICT OF INTEREST STATEMENT

The authors declare no conflicts of interest related to this study.

## Supporting information


Figure S1.

Figure S2.

Figure S3.

Figure S4.

**Data S1.** Supporting Information

## Data Availability

Data that supports the findings of this study is available in the supplementary material of this article. RNA‐sequencing data can be found on the NIH Gene Expression Omnibus website located with geo accession code GSE224900.
