# Supplementary figures and images for: Disuse‐induced muscle fibrosis, cellular senescence, and senescence‐associated secretory phenotype in older adults are alleviated during re‐ambulation with metformin pre‐treatment
Source: Aging Cell. 2023 Jul 24;22(11):e13936. doi: 10.1111/acel.13936 (PMC10652302; doi:10.1111/acel.13936)

## Supplemental Figure 1.

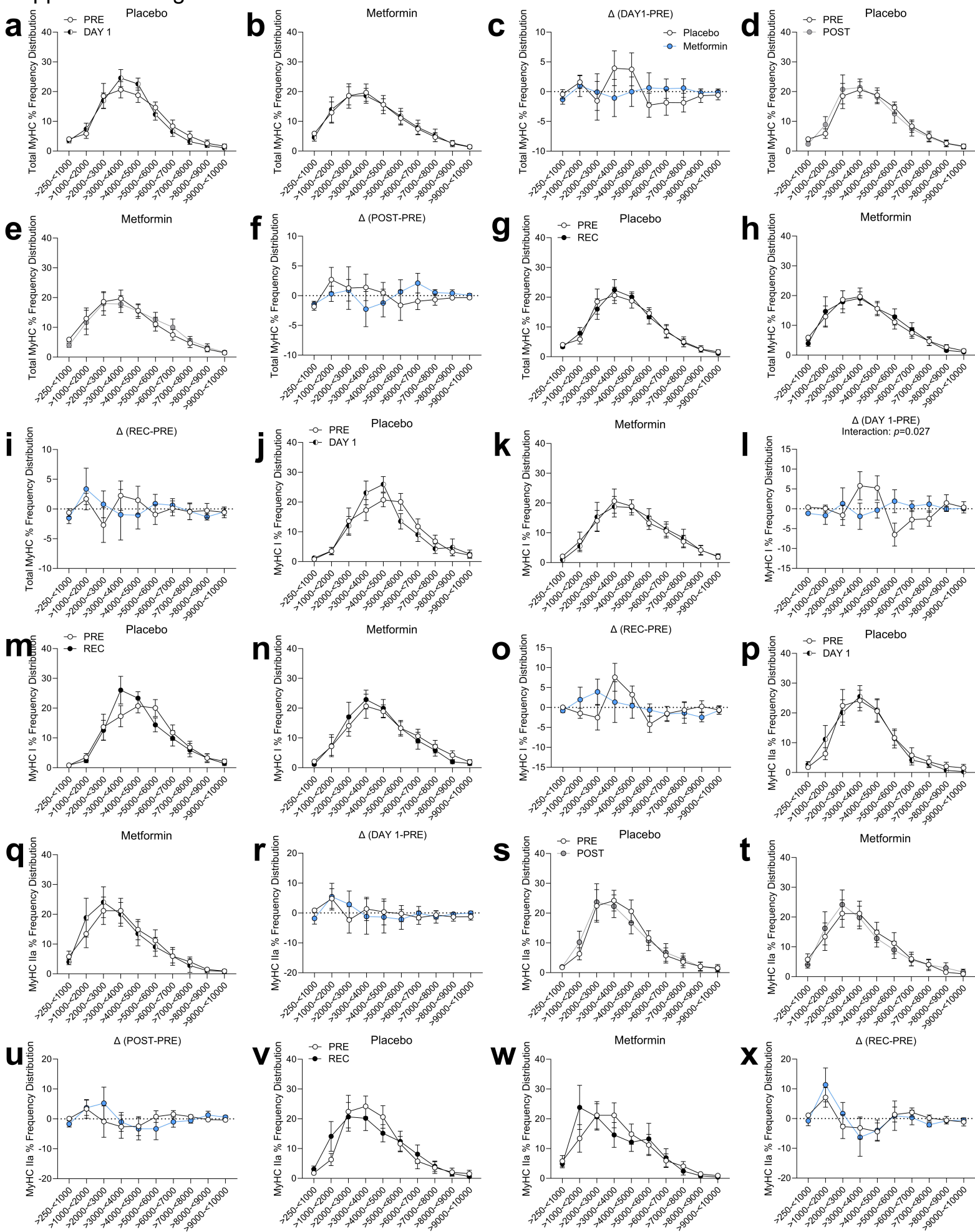

**Myofiber CSA ( $\mu\text{m}^2$ )**

Supplement: Supplementary file 1 — Figure S1. [file ACEL-22-e13936-s004.pdf]

# Supplemental Figure 2.

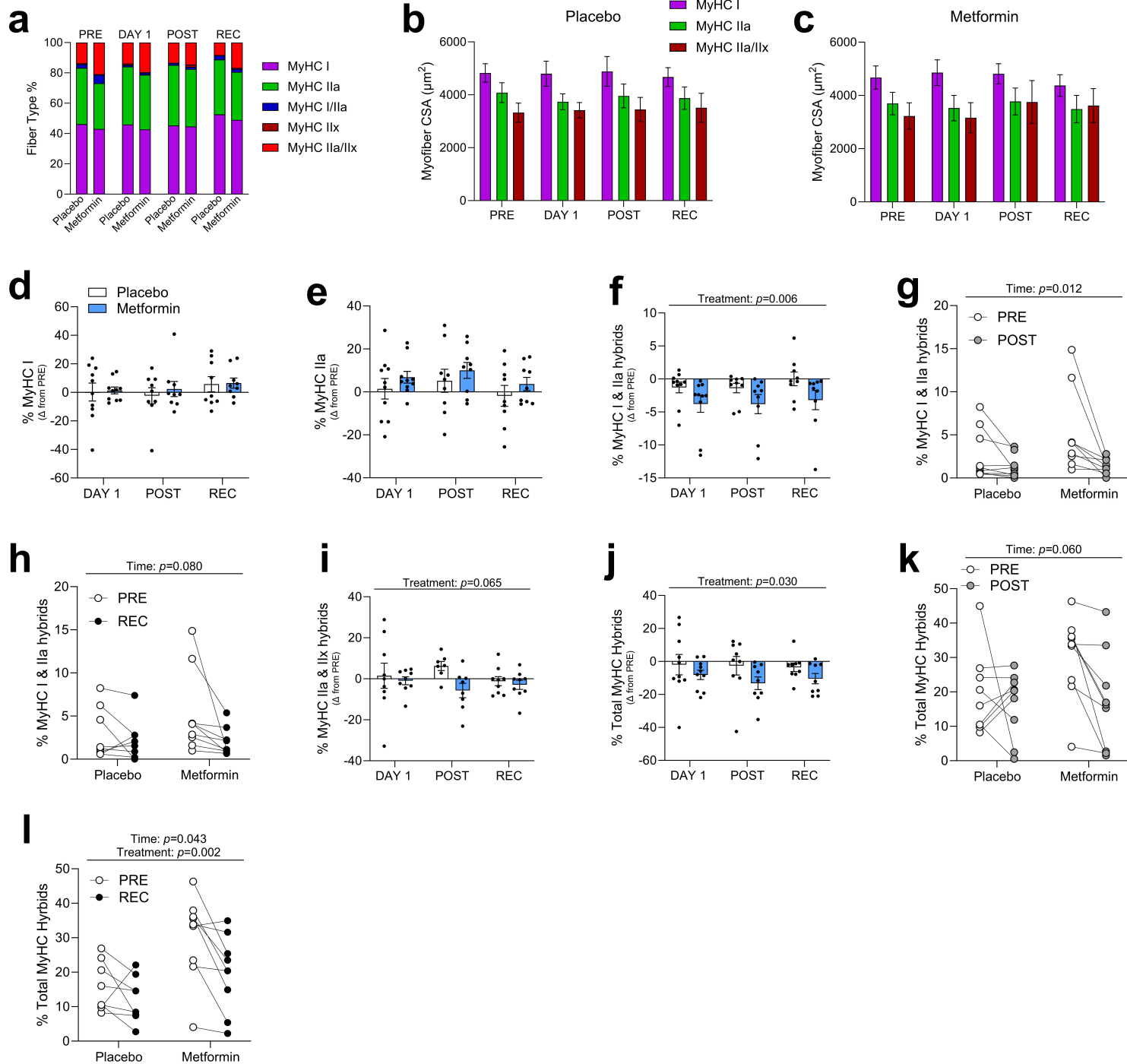

Supplement: Supplementary file 2 — Figure S2. [file ACEL-22-e13936-s005.pdf]

# Supplemental Figure 3.

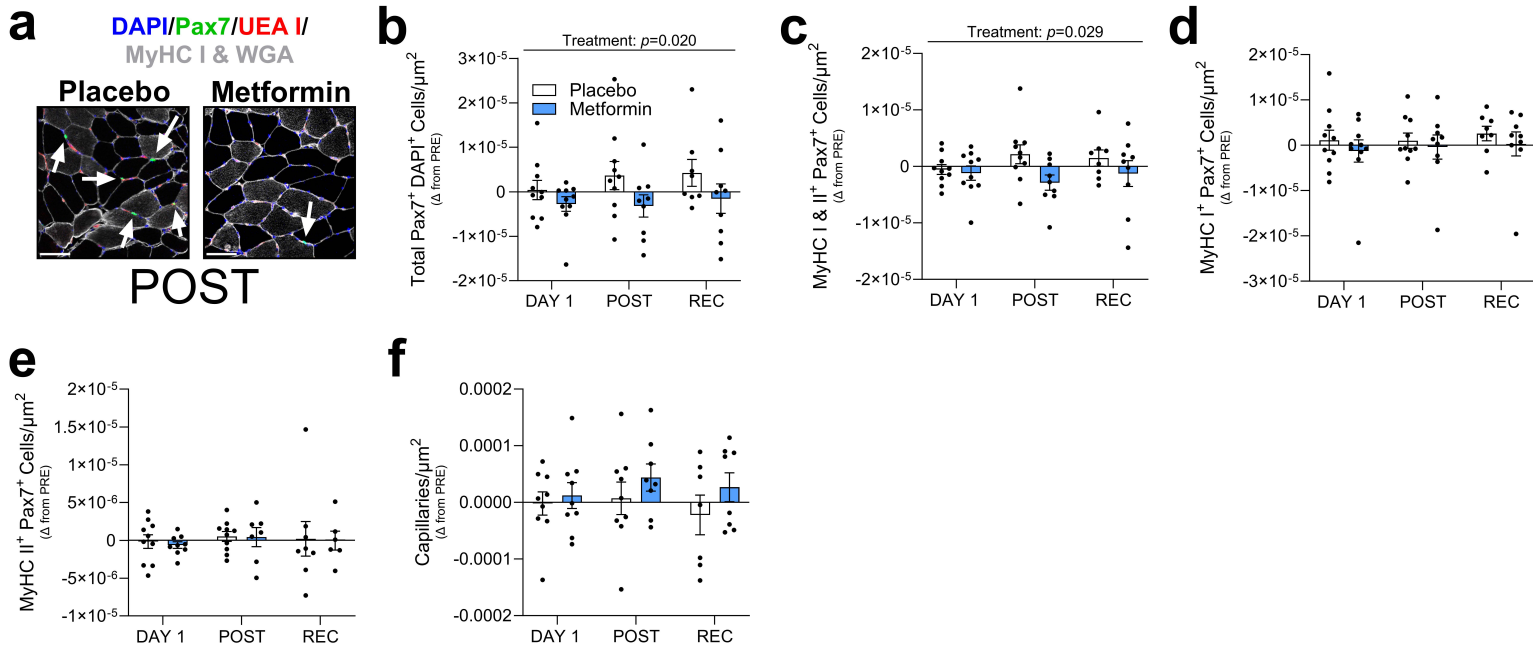

Supplement: Supplementary file 3 — Figure S3. [file ACEL-22-e13936-s001.pdf]

## Supplemental Figure 4.

**a**

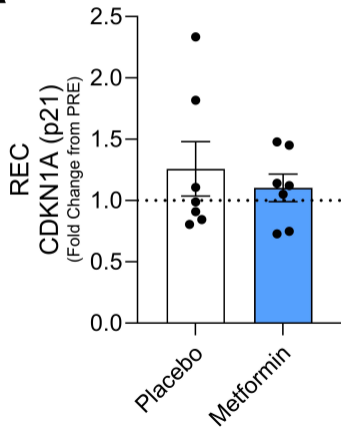

**b**

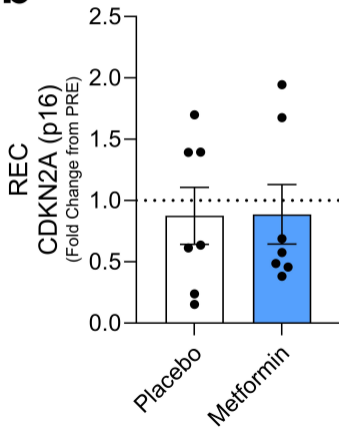

Supplement: Supplementary file 4 — Figure S4. [file ACEL-22-e13936-s002.pdf]
