# Supplementary material for: Disuse‐induced muscle fibrosis, cellular senescence, and senescence‐associated secretory phenotype in older adults are alleviated during re‐ambulation with metformin pre‐treatment
Source: Aging Cell. 2023 Jul 24;22(11):e13936. doi: 10.1111/acel.13936 (PMC10652302; doi:10.1111/acel.13936)
Supplement: Supplementary file 5 — Data S1. Supporting Information [file ACEL-22-e13936-s003.docx]

**Supplemental Methods**

*Run-in and placebo and metformin treatment*

At least 2 weeks separate the PRE-assessments from the first day of bed rest. This treatment incremental “run-in” period is common among metformin trials [1-4]. During the run-in period, participants consume incrementally increasing doses of the treatment (described below) that they are assigned and are instructed to take the capsules with a meal, to mitigate any potential GI distress symptoms, although, GI distress symptoms have been reported to subside after several days [5]. Placebo participants follow the same treatment schedule and capsule amount as the metformin group. Participants took one capsule (500 mg) with breakfast and one capsule with dinner (equaling 1 gram per day) for the first four days of the run-in. On days 5 through 9, participants took one capsule with breakfast and two capsules with dinner (1.5 grams per day). From the 10th day to the 14th day of the run-in, participants took two capsules with breakfast and two capsules with dinner (2 grams per day) and continued this dosage from the first to the fourth day of bed rest. Participants were instructed to withhold taking the treatment medication prior to the data collection procedures on the mornings of the first and fourth days of bed rest and therefore took the scheduled capsule after the data collection on those days, as to avoid a transient effect of metformin on dependent measures. Lastly, participants consumed a dose of the treatment medication on the fifth day of bed rest.

*Nutritional and physical activity assessment*

Shortly following eligibility verification participants were given specific instructions to record their dietary intake over 3 consecutive days (including 1 weekend day). Participants are also provided a pedometer and an additional record sheet to record their steps taken each day for at least 7 days prior to the bed rest visit. Caloric intake was calculated using the Food Processor Nutrition Analysis software. Participants were provided a standardized dinner based on their body weight (using the Harris-Benedict equation) to be consumed the evenings prior to muscle biopsies at PRE, DAY 1 and POST bed rest. All meals distributed throughout the course of the study consisted of controlled caloric and macronutrient density (15% protein, 55% carbohydrate, and 30% fat) diet based on the participants’ body weight. Investigators received verbal verification from the participants regarding whether they consumed the meal or not.

*Bed rest safety*

To prevent the formation of blood clots (per standard of care for hospitalized individuals), participants were provided fitted compression stockings to be worn on all 5 days of bed rest. Additionally, CCTS nurses assisted the participants in intermittently wearing pneumatic compression sleeves (2 hours per day). Daily safety blood samples were determined on the morning of each day of bed rest (PT, PTT, CBC and CMP). Also, passive range of motion movements and active neck stretches were administered daily (once per day, 20 mins per visit) by a member of the investigational team.

*Thigh and leg muscle volume*

Magnetic resonance imaging (MRI) was used for determination of muscle volume at PRE and POST. Bilateral, coronal, MRI scans (5mm thick slices separated by 5mm) of the thighs and lower leg were obtained from participants in a supine position, using a 3.0 Tesla whole body MR imager (Siemens Trio, Siemens Medical, Erlangen, Germany). Thigh axial images were analyzed starting from images immediately proximal to the patella and concluded at the image not containing the gluteus maximus tendon. Area of interest measurements were performed using the magnetic lasso tool (Adobe Photoshop CC 2019, San Jose, CA, USA). Area of interest volume was quantified by calculating the average of tracings done in triplicate on each image, multiplied by the thickness of the image taken, with the volumes from each image summed together (∑ [areas of interest x 10 mm] = area of interest volume mm^3^).

*Immunofluorescence and histochemical Analysis*

Fiber type-specific satellite cell content and muscle capillarity identification were performed as follows: Slides were fixed in cold (-20°C) acetone for 3 min, washed three times for 3 min each using 1X phosphate buffered saline (PBS), incubated with 3% H_2_O_2_ for 7 min at room temperature, washed three times for 3 min each in PBS, blocked for 1h at room temperature in 2.5% normal horse serum (NHS) and with avidin (four drops of avidin per mL of NHS, Vector Laboratories, Burlingame, CA, USA, cat# SP-2001). Slides were then incubated with primary antibodies (MyHC-1, BA.D5c, 1:100, Developmental Studies Hybridoma Bank, IA, USA; laminin, L9393, 1:200, Sigma‐Aldrich, St. Louis, MO, USA; PAX-7, Pax7c, 1:100, Developmental Studies Hybridoma Bank, IA, USA), biotin (four drops of biotin per mL of NHS, Vector Laboratories, Burlingame, CA, USA, cat# SP-2001), and capillary reagent (Rhodamine labeled Ulex Europaeus Agglutinin I, 1:50, Vector Laboratories, Burlingame, CA, USA, cat# RL-1062), in 2.5% NHS for 1h at room temperature and then overnight at 4°C. On the following day, slides were washed three times for 5 min each in PBS and incubated in the appropriate secondary antibodies in 2.5% NHS for 1h at room temperature (MyHC-1, AF647, #A21242, 1:500, Invitrogen, Carlsbad, CA, USA; laminin, AF647, #A21245, 1:500, Invitrogen, Carlsbad, CA, USA; Biotin-SP-conjugated, #115-065-205, 1:1000, Jackson ImmunoResearch, West Grove, PA, USA). Slides were then washed three times for 3 min each in PBS and then incubated in horse-radish-peroxidase conjugated to streptavidin (SA-HRP) (Tyramide SuperBoost Kit, ThermoFisher Scientific, Waltham, MA, USA, cat# B40932) for 1h at room temperature. Slides then underwent three washes for 10 min each in PBS, followed by incubation in tyramide 488 for 5 mins, according to manufacturer’s instructions. Slides then underwent one final 3-min wash three times in PBS before being mounted with DAPI containing media (Vector Laboratories, Newark, CA, USA, cat# H-1200) to then have a coverslip applied. Slides were imaged using an Axio Scan.Z1 (Zeiss, Jena, Germany) attached with an X-Cite 120 LED Boost fluorescent laser (Excelitas Technologies, Waltham, MA, USA) with a 20X objective. Myofiber type-specific satellite cell content will be assessed as described previously [7] and in accordance with Mackey, et al [8].

Myofiber type-specific staining to ascertain cross-sectional area was performed as follows: slides were rehydrated in PBS for 3 min, blocked 1h in 2.5% NHS, washed two times for 2 min each in PBS, and then incubated overnight at 4°C in primary antibodies diluted in 2.5% NHS (laminin, #L9393, 1:200, Sigma‐Aldrich, St. Louis, MO, USA; MyHC-1, BA-D5c, 1:100, MyHC-2a, SC-71c, 1:100, MyHC-2x, 6H1-s 1:10; Developmental Studies Hybridoma Bank, IA, USA). The next day, slides were washed three times for 5 min each in PBS, then incubated in the appropriate secondary antibodies (MyHC-2x, AF555, #A21426, 1:500, MyHC-1, AF647, 1:250, #A21242, MyHC-2a, AF488, #A21121, 1:500, Invitrogen, Carlsbad, CA, USA; laminin, AMCA, 1:250, #CI-1000, Vector Laboratories, Newark, CA, USA) for 1h at room temperature. Slides were then washed three times for 5 min each in PBS and then underwent a post fixation in methanol for 5 min at room temperature. Finally, slides were washed three times for 2 min each in PBS, mounted with media (#H-1000, Vector Laboratories, Burlingame, CA, USA), and then have a coverslip applied. Slides were imaged using an Axio Scan.Z1 (Zeiss, Jena, Germany) attached with an X-Cite 120 LED Boost fluorescent laser (Excelitas Technologies, Waltham, MA, USA) with a 20X objective. Myofiber type-specific myofiber cross-sectional area will be assessed as done previously [9, 10] using a semiautomated MATLAB application [11].

Collagen I and biotinylated-collagen hybridizing peptide (B-CHP; degraded collagen), staining was performed as follows: cross sections were fixed in cold (-20°C) acetone, blocked with streptavidin and biotin (Vector, San Diego, CA, USA, cat# SP-2001), and incubated in 15 μM B-CHP (3-Helix, Salt Lake City, UT, USA) following manufacturer instructions. Primary collagen I antibody (Abcam, Cambridge, UK, cat# ab34710) was added 1:100 with 15 µM B-CHP and incubated for 1h at room temperature, followed by an overnight incubation at 4°C. Dylight 594 1:200 (ThermoFisher, Waltham, MA, USA, cat# SA-5549) and secondary antibody Alexa Fluor 647 1:250 (Invitrogen, Carlsbad, CA, USA, cat# 21245) were utilized for B-CHP and collagen I, respectively. Slides were imaged using an Axio Scan.Z1 (Zeiss, Jena, Germany) attached with an X-Cite 120 LED Boost fluorescent laser (Excelitas Technologies, Waltham, MA, USA) with a 20X objective. B-CHP and collagen I were analyzed using Nikon Elements Advanced Research (Nikon, Tokyo, Japan) software to determine percent area stained. The area of collagen I and B-CHP was determined based off per channel thresholds determined using PRE group sections.

Sirius Red staining was performed as follows: muscle sections were fixed in Bouin’s solution (Labchem, Zelienople, PA, USA, cat# LC117901) for 1h at 56°C. After washing, muscles were incubated in Picro Sirius Red solution (Abcam, Cambridge, UK, cat# ab246832) for 40 min at room temperature. Muscles were then rinsed in 0.5% glacial acetic acid, then dehydrated via incubation in 90, 95 and 100% ethanol for 20s each. After drying, the sections were then quickly dipped in xylene and mounted to glass cover slips using Cytoseal XYL (ThermoScientific, Waltham, MA, USA, cat# 83124). Slides were imaged using an Axio Scan.Z1 (Zeiss, Jena, Germany) attached with an X-Cite 120 LED Boost fluorescent laser (Excelitas Technologies, Waltham, MA, USA) with a 20X objective. Sirius Red was analyzed using Nikon Elements Advanced Research software (Nikon, Tokyo, Japan). Sirius Red stained area percentage was determined using color thresholding and the area stained was outlined using region of interest tools. The threshold for Sirius Red was determined using PRE group sections. Sirius Red stained tissue was also imaged under polarized light to quantify tightly, intermediately, and loosely packed collagen on a Zeiss AxioImager M2 as published previously [12].

Second Harmonic Generation (SHG) microscopy was conducted on a Zeiss LSM 880 Upright Multiphoton Microscope fit with an Insight X3 extended wavelength laser om 20 µm thick sections. A 20X water immersion objective was used in conjunction with the multiphoton laser, tuned to 830 and 890nm. Five random fields of view were imaged to obtain image stacks with a total thickness of 20μm and slice thickness of 1μm. Images were analyzed in ImageJ with FibrilTool, to quantify fibrillar structures in raw microscopy images.

Tcf4 tissue staining was performed as follows: slides were fixed in 4% paraformaldehyde (PFA) followed by three washes at 3 min each in 1X PBS. Antigen retrieval with 10 mM sodium citrate buffer at 6.5 pH was performed at 65°C with a gradual increase to 92°C over the course of 20 min, then cooled to room temperature. After 1X PBS washes, slides were blocked in 1% BSA in PBS for 1h and then incubated overnight at room temperature in primary antibody Tcf4 1:100 (Cell Signaling Technologies, Danvers, MA, USA, cat# 2569) in 1% BSA. After four washes at 5 minutes each in 1X PBS, slides were incubated at room temperature for 85 min in goat anti rabbit secondary antibody 1:1000 (Jackson ImmunoResearch, West Grove, PA, USA, cat# 111-065-003) in 1% BSA. After PBS washes, endogenous peroxidases were blocked with 3% H_2_O_2_ in PBS for 7 min. Slides were washed and then incubated in SA-HRP for 1h at room temperature from the Tyramide SuperBoost Kit (ThermoFisher, Waltham, MA, USA, cat# B40932). Following the Tyramide SuperBoost Kit instructions, slides were incubated for 15 min in Alexa Fluor 488 amplification reagents. After PBS washes, slides were mounted in media containing DAPI (Vector Laboratories, Newark, CA, USA, cat# H-1200) and imaged on an Axio Scan.Z1 (Zeiss, Jena, Germany) attached with an X-Cite 120 LED Boost fluorescent laser (Excelitas Technologies, Waltham, MA, USA) with a 20X objective. Images were analyzed using Nikon Advanced Research Elements software with thresholds for Tcf4 and DAPI using PRE group sections. Total myofiber area was determined and the number of Tcf4+ and DAPI+ cells was divided by the total area of the image.

Macrophage staining was performed as in [13] and briefly as follows: Sections were fixed in -20°C acetone at room temperature followed by PBS washes. Sections were incubated for 8 min with 3% H_2_O_2_ in PBS at room temperature and then PBS washed. Slides were then incubated for 1h at room temperature in 2% NHS with vector avidin D solution (4 drops 1 mL) and primary antibody CD11b 1:100 (Cell Sciences, Newburyport, MA, USA, cat# MON1019-1). After washes, slides were incubated for 30 min at room temperature in secondary donkey anti mouse antibody 1:500 (Jackson ImmunoResearch, West Grove, PA, USA, cat# 715-065-150) in 2.5% NHS. Following PBS washes, sections were incubated in SA-HRP from Tyramide SuperBoost Kit (ThermoFisher, Waltham, MA, USA, cat# B40932) for 30 minutes at room temperature and then in Alexa Fluor 488 for 10 minutes with SuperBoost kit amplification reagents. After washes, sections were incubated in 2.5% NHS with vector avidin D for 10 min at room temperature. Next, sections were incubated overnight in CD206 1:200 (R&D Systems, Minneapolis, MN, USA, cat# AF2534) in 2.5% NHS at 4°C. Following washes, sections were incubated for 1h at room temperature with secondary Cy3 antibody 1:250 (Jackson ImmunoResearch, West Grove, PA, USA, cat# 805-165-180). After washes, sections were incubated in 647 WGA 1:50 (Invitrogen, Waltham, MA, USA, cat# W32466) in 1X PBS and mounted in media containing DAPI (Vector Laboratories, Newark, CA, USA, cat# H-1200). Images were acquired with on an Axio Scan.Z1 (Zeiss, Jena, Germany) attached with an X-Cite 120 LED Boost fluorescent laser (Excelitas Technologies, Waltham, MA, USA) with a 20X objective. Images were analyzed using Nikon Advanced Research Elements software with thresholds for CD11b, CD206, and DAPI using PRE group sections. Total myofiber area was determined and the number of CD11b+, CD206+, and DAPI+ cells was divided by the total area of the image.

Senescence associated (SA)-β-galactosidase staining was performed according to the manufacturer’s instructions (Cell Signaling Technology, cat# 9860). Briefly, cells were washed 3 times with 1x PBS and fixed for 15 minutes at room temperature. After two 1x PBS washes, a β-galactosidase staining solution containing a final concentration of 1 mg/mL X-gal and a pH of 6.0 was added to the cells, the culture plate was sealed in parafilm and incubated overnight at 37°C in a dry incubator (no CO_2_). The β-Galactosidase staining solution was removed and replaced with 70% glycerol. Images were taken at 10X magnification on a wide-field light microscope (Nikon Ti, Tokyo, Japan) utilizing a Nikon DS-Ri2 camera.

Alpha-smooth muscle actin (α-SMA) staining was performed in muscle resident fibroblasts fixed in warm (37°C) 4% PFA for 20 minutes, washed in 1x PBS, then permeabilized in 0.1% Triton-X100 in 1x PBS. Cells were blocked in 1% BSA for 1 h then incubated in primary Tcf4 (Cell Signaling Technologies, Danvers, MA, USA, cat# 2569) 1:100 and α-SMA (Santa Cruz, Dallas, TX, USA, cat# sc130616) 1:100 for 2h at room temperature in 1% BSA/0.1% Triton-X100. After washing, cells were incubated for 85 minutes in secondary anti rabbit biotinylated (Jackson ImmunoResearch, West Grove, PA, USA, cat# 111-065-003) antibody 1:1000 and mouse IgG2a secondary (Invitrogen, Waltham, MA, USA, cat# A-21131) 1:250 in 1%BSA/0.1% Triton-X100. After washing, cells were treated following Tyramide SuperBoost Kit (ThermoFisher, Waltham, MA, USA, cat# B40933) instructions, then incubated with DAPI for 10 minutes. Images were taken at 20X magnification on a wide-field light microscope (Nikon Ti, Tokyo, Japan) utilizing a high sensitivity Andor Clara CCD camera (Belfast, UK).

BODIPY staining was performed as follows: Cells were washed with 1x PBS followed by fixing in 4% PFA for 20 minutes at room temperature. After 1x PBS washes, cells were incubated in 10 µg/mL BODIPY (ThermoFisher, Waltham, MA, USA, cat# D3922) working solution. Cells were washed in 1x PBS and then incubated in DAPI for 10 minutes. Images were taken at 60X magnification on a wide-field light microscope (Nikon Ti, Tokyo, Japan) utilizing a high sensitivity Andor Clara CCD camera (Belfast, UK).

**References**

1. Konopka, A.R., et al., *Metformin inhibits mitochondrial adaptations to aerobic exercise training in older adults.* Aging Cell, 2019. **18**(1): p. e12880.

2. Long, D.E., et al., *Metformin to Augment Strength Training Effective Response in Seniors (MASTERS): study protocol for a randomized controlled trial.* Trials, 2017. **18**(1): p. 192.

3. Malin, S.K., et al., *Independent and combined effects of exercise training and metformin on insulin sensitivity in individuals with prediabetes.* Diabetes Care, 2012. **35**(1): p. 131-6.

4. Walton, R.G., et al., *Metformin blunts muscle hypertrophy in response to progressive resistance exercise training in older adults: A randomized, double-blind, placebo-controlled, multicenter trial: The MASTERS trial.* Aging Cell, 2019. **18**(6): p. e13039.

5. Sharoff, C.G., et al., *Combining short-term metformin treatment and one bout of exercise does not increase insulin action in insulin-resistant individuals.* Am J Physiol Endocrinol Metab, 2010. **298**(4): p. E815-23.

6. Evans, W.J., S.D. Phinney, and V.R. Young, *Suction applied to a muscle biopsy maximizes sample size.* Med Sci Sports Exerc, 1982. **14**(1): p. 101-2.

7. Reidy, P.T., et al., *An accumulation of muscle macrophages is accompanied by altered insulin sensitivity after reduced activity and recovery.* Acta Physiol (Oxf), 2019. **226**(2): p. e13251.

8. Mackey, A.L., et al., *Assessment of satellite cell number and activity status in human skeletal muscle biopsies.* Muscle Nerve, 2009. **40**(3): p. 455-65.

9. Reidy, P.T., et al., *Neuromuscular Electrical Stimulation Combined with Protein Ingestion Preserves Thigh Muscle Mass But Not Muscle Function in Healthy Older Adults During 5 Days of Bed Rest.* Rejuvenation Res, 2017. **20**(6): p. 449-461.

10. Petrocelli, J.J., et al., *Metformin and leucine increase satellite cells and collagen remodeling during disuse and recovery in aged muscle.* FASEB J, 2021. **35**(9): p. e21862.

11. Smith, L.R. and E.R. Barton, *SMASH - semi-automatic muscle analysis using segmentation of histology: a MATLAB application.* Skelet Muscle, 2014. **4**: p. 21.

12. Brightwell, C.R., et al., *Muscle fibrosis and maladaptation occur progressively in CKD and are rescued by dialysis.* JCI Insight, 2021. **6**(24).

13. Reidy, P.T., et al., *Neuromuscular electrical stimulation and protein during bed rest increases CD11b(+) skeletal muscle macrophages but does not correspond to muscle size or insulin sensitivity.* Appl Physiol Nutr Metab, 2020. **45**(11): p. 1261-1269.
